# Supplementary material for: Study protocol for a pilot randomised controlled trial evaluating the feasibility and effectiveness of non-pharmacological interventions to recover functionality after a transient ischaemic attack or a minor stroke: the ‘Back to Normal’ trial
Source: BMJ Open. 2023 Apr 28;13(4):e069593. doi: 10.1136/bmjopen-2022-069593 (PMC10151926; doi:10.1136/bmjopen-2022-069593)
Supplement: Author's manuscript [file bmjopen-2022-069593.draft_revisions.pdf]

BMJ Open is committed to open peer review. As part of this commitment we make the peer review history of every article we publish publicly available.

When an article is published we post the peer reviewers' comments and the authors' responses online. We also post the versions of the paper that were used during peer review. These are the versions that the peer review comments apply to.

The versions of the paper that follow are the versions that were submitted during the peer review process. They are not the versions of record or the final published versions. They should not be cited or distributed as the published version of this manuscript.

BMJ Open is an open access journal and the full, final, typeset and author-corrected version of record of the manuscript is available on our site with no access controls, subscription charges or pay-per-view fees (http://bmjopen.bmj.com).

If you have any questions on BMJ Open's open peer review process please email info.bmjopen@bmj.com

BMJ Open

A study protocol for a pilot randomised controlled trial evaluating the feasibility and effectiveness of non-pharmacological interventions to recover functionality after a transient ischemic attack or a minor stroke: the "Back to Normal" trial

Journal:	*BMJ Open*
Manuscript ID	bmjopen-2022-069593
Article Type:	Protocol
Date Submitted by the Author:	14-Nov-2022
Complete List of Authors:	Lopes Gonçalves, Micaela; University of Porto Epidemiology Research Unit<br>Lima , Maria João; Unidade Local de Saúde de Matosinhos EPE, Neurology<br>Fonseca, Ângelo ; Unidade Local de Saúde de Matosinhos EPE, Neurology<br>Duque, Cristina; Unidade Local de Saúde de Matosinhos EPE, Neurology<br>Costa, Ana ; University of Porto Epidemiology Research Unit; FMUP, Departamento de Ciências da Saúde Pública e Forenses e Educação Médica<br>Cruz, Vitor; University of Porto Epidemiology Research Unit; Unidade Local de Saúde de Matosinhos EPE, Neurology
Keywords:	Stroke < NEUROLOGY, PUBLIC HEALTH, PREVENTIVE MEDICINE

SCHOLARONE™
Manuscripts

A study protocol for a pilot randomised controlled trial evaluating the feasibility and effectiveness of non-pharmacological interventions to recover functionality after a transient ischemic attack or a minor stroke: the "Back to Normal" trial

Micaela Gonçalves[a,b], Maria João Lima[c], Ângelo Fonseca[c], Cristina Duque[c], Ana Rute Costa[a,b,d], Vítor Tedim Cruz[a,b,c]

[a] EPIUnit – Instituto de Saúde Pública, Universidade do Porto, Rua das Taipas, n° 135, 4050-600 Porto, Portugal

[b] Laboratório para a Investigação Integrativa e Translacional em Saúde Populacional (ITR), Universidade do Porto, Rua das Taipas, n° 135, 4050-600 Porto, Portugal

[c] Serviço de Neurologia, Hospital Pedro Hispano, Unidade Local de Saúde de Matosinhos, Rua Dr. Eduardo Torres, 4464-513 Senhora da Hora, Portugal.

[d] Departamento de Ciências da Saúde Pública e Forenses e Educação Médica, Faculdade de Medicina da Universidade do Porto, Alameda Professor Hernâni Monteiro, 4200-319 Porto, Portugal.

Corresponding author:

Micaela Gonçalves

Instituto de Saúde Pública da Universidade do Porto (ISPUP)

Rua das Taipas, nº 135

4050-600 Porto, Portugal

Telephone: +351 222061820

E-mail: micaela.goncalves@ispup.up.pt

For peer review only

Abstract

Introduction: Transient ischemic attack (TIA) and minor stroke are frequently assumed as temporary or non-disabling events. However, evidence suggests that these patients can experience relevant impairment and functional disability. As such, the present study aims to evaluate the feasibility and effectiveness of a three-month multidomain intervention program, composed of five non-pharmacological strategies, aimed at accelerating return to pre-event level of functionality in patients with TIA or minor stroke.

Methods and Analysis: Patients diagnosed with a TIA or a minor stroke are being recruited at the emergency or neurology departments of the Hospital Pedro Hispano, located in Matosinhos, Portugal (n=70). Those who accept to participate will be randomly allocated to two groups (1:1): (a) Intervention – receives a three-months combined approach, initiating early post-event, composed of cognitive training, physical exercise, nutrition, psychoeducation and assessment/correction of hearing loss; (b) Control – participants will not be subject to any intervention. Both groups will receive the usual standard of care provided to these diseases. Recruitment began in May 2022 and is expected to continue until December 2022. Sociodemographic characteristics, lifestyles, health status, cognitive function, symptoms of anxiety and depression, and quality of life will be assessed; as well as anthropometry, blood pressure and physical condition. The time to complete or partial recovery of instrumental activities of daily living will be assessed using an adapted version of the Frenchay Activities Index. All participants will be evaluated before the intervention and 3 months after the beginning of the intervention.

Ethics and Dissemination: This study was approved by the hospital's Ethics Committee. Written informed consent will be required from all the participants; data protection and confidentiality

For peer review only - http://bmjopen.bmj.com/site/about/guidelines.xhtml

will be also ensured. The findings of this project are expected to be submitted for publication in scientific articles, and the main results will be presented at relevant scientific meetings.

Trial Registration Number: NCT05369637

Keywords: Ischemic Attack, Transient; Minor Stroke; Non-Pharmacological Interventions; Activities of Daily Living; Randomised Controlled Trial

Word Count: 3665

For peer review only

Strengths and Limitations of this study

- This protocol describes a pilot randomized controlled trial addressing the effect of an innovative multidomain non-pharmacological intervention program on post-transient ischemic attack and minor stroke functionality.
- The intervention program will start early after the diagnosis of the vascular event, maximizing health gain and targeting brain plasticity.
- The main outcome is based on the time to recover instrumental activities of daily living after the event, which is more suitable due to the characteristics of residual impairment.
- This study will explore a wide range of different outcomes, including study feasibility, cognitive function, lifestyle behaviors, physical and emotional status.
- Given the characteristics of the intervention, blinding of participants is not possible, the main outcome is based on a pre-defined list of activities of daily living and will be self-reported, being prone to recall bias.

For peer review only

Introduction

A Transient Ischemic Attack (TIA) is defined as a “transient episode of neurological dysfunction caused by focal brain, spinal cord, or retinal ischemia, without acute infarction” by the American Heart Association,[1] while a minor stroke is suggested to occur when a patient has a score of ≤3 in the National Institutes of Health Stroke Scale (NIHSS).[2] Population-based studies have shown that the age-adjusted incidence of TIA in the European population ranges from 28 to 59/100 000/year[3] and, in Portugal, a higher annual TIA incidence of 74/100 00/ people was estimated between 2009–2011.[4] This is particularly relevant since previous studies have described an increased risk of stroke after these events,[5] and, in 2019, stroke was the third leading cause of disability-adjusted life years (DALYs).[6]

The study of the overall morbidity associated with a TIA or a minor stroke is often neglected, since most of the time they are assumed as being transient or non-disabling events. However, evidence suggests that these patients can experience relevant disability even with a NIHSS of 0 or 1.[7] A decreased performance in activities of daily living (ADLs) up to 1-year post-event was also observed, including in patients without any apparent physical disability.[8,9] Other studies showed a high prevalence of depression and apathy,[10] cognitive impairment,[11] physical deficits[12] and hearing degeneration,[13] with implications on patients’ return to work, social relations and activities.[14] Moreover, European guidelines on management of TIA recommend for early initiation of secondary prevention following such an event,[15] focusing particularly on the leading risk factors for stroke, namely high systolic blood pressure, body mass index, fasting plasma glucose and smoking.[6]

Taken together, these findings highlight the importance of multidomain interventions aiming to promote functioning levels among patients with TIA or minor stroke, and to develop preventive

For peer review only

actions and health promotion plans specifically designed for this group. As such, this pilot study primarily aims to explore the feasibility and the effectiveness of an innovative multidomain intervention program, composed of five strategies: cognitive training, physical exercise, nutrition education, psychoeducation and assessment/correction of hearing problems. These are aimed at accelerating the return to pre-event functionality in patients with a TIA or a minor stroke diagnosis. The primary objectives are as follows:

1) To assess the feasibility of methodology for early recruitment, collecting information and delivering the interventions;

2) To estimate the effectiveness of this program on the time to recovery in each instrumental ADLs.

As secondary objectives, the effect of this intervention on cognitive function, adherence to the Mediterranean diet, physical function, basic ADLs, symptoms of anxiety and depression, quality of life, anthropometric measures and blood pressure, will be also assessed.

For peer review only

Methods and Analysis

Study design

The present study is a two-arm parallel, superiority, pilot randomised controlled trial (RCT), which will be nested in the ongoing project “Multiple Interventions to Prevent Cognitive Decline” (MIND-Matosinhos).[16] The primary endpoints will be the feasibility of this multidomain program, as well as the time to recovery to pre-event functionality after the occurrence of a TIA or a minor stroke. A block randomisation with a 1:1 allocation will be performed.

Setting

This pilot RCT will be conducted at the department of neurology of the Hospital Pedro Hispano E.P.E (HPH), as part of the Local Health Unit of Matosinhos (ULSM), which is integrated in the Portuguese National Health Service. The HPH is located at the Matosinhos municipality, Northern Portugal, and is responsible for providing care to 318 000 inhabitants from the municipalities of Matosinhos, Vila do Conde and Póvoa de Varzim.

Eligibility criteria

Patients aged 18-85 years old and with a clinical diagnosis of TIA or minor stroke as defined by a National Institute of Health Stroke Scale (NIHSS) score ≤3 are eligible for this study. Additional inclusion criteria include: the onset of symptoms within the last seven days; first-time stroke or TIA; at least four or more years of education; discharged home without the need for inpatient rehabilitation; modified Rankin Scale (mRS) 0 to 2. Patients will be excluded if they are unable to attend the face-to-face intervention sessions, or if they have: previous diagnosis of dementia

For peer review only - http://bmjopen.bmj.com/site/about/guidelines.xhtml

or severe disability; contraindication for physical exercise, severe loss of hearing, vision, or communication skills; frailty, reduced life expectancy due to severe disease or need for regular treatments that compete with availability for intervention (e.g., chemotherapy, haemodialysis).

Recruitment

Participants will be enrolled through consecutive sampling from the Neurology Department (Stroke or Emergency care units) of HPH. Healthcare professionals from the recruitment site will be informed about the protocol and their potential role, and an experienced researcher will be on-site to provide coordination. The Neurology medical team will be responsible for screening eligibility and obtaining written informed consent for all the participants. Recruitment started on May 11, 2022.

Randomization

The participants will be randomly assigned (1:1) to the intervention or control group after the baseline assessment. Randomization will be done using a computer-generated program, using random blocks of four sizes. A researcher who is not immediately engaged in the project will implement the randomization process.

For peer review only

Data collection

Participants will be evaluated at baseline (before the beginning of the intervention program) and at three months, as depicted in Figure 1. Data on sociodemographic (birth date, sex, address, marital status, education, occupation, income), medical history, current medication, lifestyle (smoking and alcohol use, physical activity and sedentary behaviors) and adherence to Mediterranean diet [17] will be collected through structured questionnaires applied by a trained researcher. Anxiety and depression,[18] memory complaints [19] and quality of life [20] will be evaluated through self-administered questionnaires validated for the Portuguese population. Additionally, long term cognition data will be also collected every 3 months for a period of up to 10 years using Brain on Track® (BoT), a web-based tool for remote longitudinal assessment of different cognitive domains.[21] The outcome measure section describes the instruments in detail.

Data regarding clinical diagnosis, onset of symptoms, hospital admission and discharge date, mRS score, NIHSS score, medical history of cerebrovascular events, age, and need for inpatient/outpatient rehabilitation will be retrieved from the patient´s medical record. Clinical characteristics of the cerebrovascular event will also be collected.

Intervention

The intervention will be implemented over three months and will comprise five different components, including cognitive training, physical exercise, nutritional education, psychoeducation and assessment and correction of hearing problems. Only the intervention group will be exposed to all or any of the components of this comprehensive intervention program. Both groups will continue to receive the usual standard of care provided to this type of clinical disease in the hospital setting. The usual standard of care consists of the initiation of

For peer review only - http://bmjopen.bmj.com/site/about/guidelines.xhtml

the appropriate secondary prevention and referral to the outpatient cerebrovascular diseases clinic (telephone call at 1-month post-event and in-person appointment at 3-months post-event). Depending on the clinical presentation and neurological deficits, patients can also be referred to an outpatient physical medicine and rehabilitation appointment.

Since early intervention post-vascular event is decisive, given the time interval between neurological injury and the onset of nervous function recovery activities, intervention sessions are aimed to begin within no later than 15 days after the cerebrovascular event. Considering this timeline, once the participants are recruited and randomized, the intervention will start immediately, especially cognitive training.

To minimize refusals and losses to follow-up, all evaluations and sessions will be scheduled by the participant's preference. A written plan with the intervention schedules will be given and sessions reminded through email or phone messages in the day before (day, time, and location). If one intervention session is missed, efforts will be placed to arrange another suitable time in another group, and individualized meetings will be rescheduled. If not possible, the missed appointment will be documented, and the dropout or non-compliance reasons will be registered for further analysis.

Cognitive training

Monthly in-person sessions in groups of a minimum of five participants (60 min) will be conducted by a psychologist. Additionally, during the three months a daily home-based cognitive training individual (30 minutes) sessions using the COGWEB® [22] online platform will be performed. This online computer tool provides cognitive training tailored to each patient's deficits with different difficulty levels, allowing continuous treatment monitoring, clinical evolution, and adherence compliance.[22] Exercises are aimed at different cognitive domains, such as attention, memory, executive function, language and calculus.[22] Participants have

For peer review only

access to instructions for each exercise before initiating the session, displayed on the screen and through the speakers. Instructions can be seen at any moment, even throughout the session. Participants without access to a computer will receive paper/pencil equivalent home-based cognitive exercises.

Physical exercise

Physical exercise sessions in-person or remotely twice a week with a duration of up to 60 minutes, guided and supervised by a graduated physical exercise technician, will be conducted. Activities will be tailored to each group's tolerance, medical history, and comorbidities, focusing on impaired balance and weakness. During exercise sessions, the exercise technician will track symptoms of chest pain, shortness of breath, wheezing, dyspnoea, light-headedness, nausea, among others. Small groups allow to set short and attainable goals with the participants and have a graded exercise program, starting with reduced intensity and gradually increasing.

In-person sessions will be preferred, however if online sessions are needed, the participants without a computer will receive booklets with physical exercise instructions twice a week and the researcher will contact the participants to assess adherence to the exercises.

Nutritional education

Intervention arm will include individual appointments (three appointments with a nutritionist, once a month with a duration of 45 min to 60min) and in-person group cooking sessions (once a month with a duration of 90 min). During individual sessions, the nutritionist will emphasize the benefits of adhering to the Mediterranean diet and salt reduction, particularly for overall cardiovascular health advantages. Group sessions start with the nutritionist's presentation of healthy recipes and further analysis of nutritional values, then the participants will undergo its preparation. In these sessions, participants can bring their partners or one family member.

For peer review only - http://bmjopen.bmj.com/site/about/guidelines.xhtml

Psychoeducation

Monthly in-person psychoeducation sessions (60 minutes) will be conducted by a psychologist. These sessions will allow the acquirement of crucial knowledge about secondary prevention of cerebrovascular events (signs and symptoms, vascular risk factors, adherence to medication) and support people in understanding, exploring, and self-managing their emotions and impairments, through peer support, and promotion of lifestyle changes.

Assessment and correction of hearing problems

At the beginning of the intervention program, a hearing assessment and correction will be performed. This evaluation will be conducted by otolaryngologists and audiologists, who will evaluate previous hearing problems and use of hearing aids, and includes otoscopy and audiogram. Those detected with any hearing problem will be referred for a medical appointment with an otolaryngologist.

Primary outcome measures

The primary outcomes for the study are related with its feasibility and time to partial or complete recovery of each instrumental ADLs.

To evaluate the feasibility of this study, the following primary outcomes will be assessed:

- Recruitment timeframe: proportion of participants recruited within seven days after onset of symptoms. It will be assessed by dividing the number of participants recruited within the time frame divided by the total number of participants.
- Beginning of the intervention: proportion of participants that started any of the interventions no later than 15 days after onset of symptoms.

For peer review only

- Adherence to each component of the intervention: proportion of adherence to each component of the intervention and different intervention modalities (remote/in person), calculated as the number of sessions attended divided by the total number of sessions provided. For the COGWEB® training, the outcome will be the absolute number of sessions.
- Dropout: proportion of participants who dropped out of the study, calculated as the number of participants who dropped out after attending at least one session divided by the total number of participants who attended at least one session.

Time to recovery in each instrumental activity of daily living will be measured by an adapted version of the Frenchay Activities Index (FAI). The translated and validated version of the FAI – adapted version for the Portuguese population [23] will be used to assess the instrumental ADLs functional performance at baseline and follow-up at three months. The version used in this study is based on the perception of the patient's capacity to perform a specific instrumental ADLs instead of its frequency, as stated in the original scale developed by Holbrook and Skilbeck.[24] The scale consists of 15 activities, scored from 1 to 4 (1-Could not do at all; 2-very difficult; 3-a little difficult; 4-not difficult at all).[23] Although the original index suggests that total scores for men and women should be considered separately,[24] in the present study the score of each activity will be analysed independently, as the interest in exploring the impact of the programme in each activity.

For the present study, only activities that are part of participants' daily lives will be considered, namely those that were performed at least once in the last 12 months. Time to recovery will be defined as the time from the start of the intervention program to the time of complete or partial recovery. Each participant will report their level of performance in each activity at the baseline and follow-up at three months. In the baseline, if a participant scores a specific activity as altered (score 1 to 3), their level of performance before the TIA or minor stroke will be confirmed to

For peer review only - http://bmjopen.bmj.com/site/about/guidelines.xhtml

understand if the change in the activity performance is related with the cerebrovascular event. It will be also inquired and recorded the date when a difficulty in a certain activity ceased. The average of activities altered post-event (score 1,2,3) at baseline and the average of activities that improved in both groups upon completing the intervention program will be measured.

Secondary outcome measures

Secondary outcomes and the instruments that will be used for their assessments are described in Table 1 and Table 2. Briefly, secondary endpoints related with the study's feasibility will be measured, namely time of follow-up, implemented sessions and complete assessment of participants (Table 1). Additionally, cognitive function, quality of life, symptoms of anxiety and depression, as well as functional capacity to perform basic ADLs will be evaluated through questionnaires validated for the Portuguese population (Table 2). Physical condition, blood pressure, and anthropometrics will be ascertained by trained interviewers. Levels of glycated haemoglobin, and 24-hour urinary excretion of sodium and potassium will be also measured.

Table 1. Description of the secondary feasibility outcomes.

OUTCOME	DESCRIPTION
**Time of follow-up**	Calculated by the number of days between the participant's first and last session attended.
**Implemented sessions**	Proportion of sessions implemented, calculated as the number of sessions that the research team was able to implement divided by the total number of sessions planned.
**Complete assessment of participants**	Proportion of participants with full information at baseline and follow-up points, calculated by dividing the number of participants with complete information by the number of participants evaluated.

For peer review only - http://bmjopen.bmj.com/site/about/guidelines.xhtml

Table 2. Description of the instruments used for the evaluation of secondary outcomes.

OUTCOME	INSTRUMENT	DESCRIPTION
**Cognitive performance**	MoCA [25–27]	Brief screening tool to detect mild cognitive impairment. It assesses eight cognitive domains: visuospatial ability, executive function, attention, concentration, working memory, language, verbal memory, and orientation, with scores from 0 to 30. Higher scores represent better cognitive performance, and one point is added for participants with less than 12 years of education.
	Brain on Track® [21,28]	Self-administered computerized cognitive test for remote longitudinal assessment. It evaluates different cognitive domains through exercises, such as attention, memory, executive functions, language, calculation, constructive capacity, and visuospatial processing. The score range is the maximum number of correct answers within a limit of time. Higher scores are representative of better cognitive performance.
**Memory Complaints**	Subjective Memory Complaints Scale [19,29]	This scale comprises 10 questions regarding subjective complaints of memory. This scale varies from 0 (best score) to 21 points (worst score).
**Adherence to the Mediterranean diet**	MEDAS questionnaire [17]	The MEDAS questionnaire is composed by 14 questions associated with eating habits and frequency of food consumption. The score ranges between 0 (lowest adherence to the Mediterranean diet) to 14 points (highest adherence to the Mediterranean diet). A score greater than 9 points demonstrates a good adherence to the Mediterranean diet.
**Anxiety and Depression**	HADS [18]	Scale with 14 questions, subdivided in two subscales: anxiety (HADS-A) and depression (HADS-D). This scale varies from 0 (best score) to 21 (worst score) for each sub-scale. The optimal cut-off points differ significantly in the literature. For this study, a score higher than or equal to 11 represents a case of anxiety or depression, as applicable.
**Reported quality of life**	EQ-5D-5L [20,30]	This scale is subdivided into two subscales: a) five multiple-choice questions, with five response options, which results in a score that fluctuates from 5 (best score) to 25 points (worst score). One digit is provided for each dimension, combining in a 5-digit code. A total of 3125 possible health states are delineated to describe the patient's state of health. b) vertical visual analogic scale, which varies from 0 (worst score) to 100 (best score).
**Disability**	Modified Rankin Scale [31,32]	Simple scale that assesses functional capacity, referring to limitations in "activity and changes in lifestyle". The scores run from 0 (perfect health) to 6 (dead). It will be considered disability as an mRS of > 1 and a one-point change on the scale will be clinically significant.
**Handgrip strength**	Dynamometer [33–35]	Force applied in each hand in kilograms using the JAMAR dynamometer. The handgrip can be a relevant predictor of the physical function of the upper body and influences the capacity to perform ADLs. Participant will be in a seated position with the elbow flexed at 90° degrees. Three alternating hand sequential measurements will be performed and registered. It is relevant to document the dominant hand and the affected and non-affected hands if the case.
**Agility**	Time UP and Go test scale [36]	It consists of counting the participant's time to complete the following routine: standing up from a chair, walking for 3 meters (following the marks on the floor), returning to the chair, and sitting down as fast as possible. It provides important information about functional capacity, gait, dynamic balance, and speed. The time will be counted from when the participant's back leaves the chair until sitting again with their back supported. It will be considered a cut-off level of ≤ 10 seconds to identify normal mobility.
	Unipedal	This test is performed in time units (seconds) and the score varies from 0

For peer review only - http://bmjopen.bmj.com/site/about/guidelines.xhtml

	stance test [37]	(worse score) to 45 seconds (best score). The researcher will use a stopwatch and count the amount of time the participant can stand on one leg, of their choice, with their eyes open.
	Senior Fitness Test [38,39]	Variation of participant's agility and dynamic balance assessed using the 8-foot distance test (2,44 meters) from the Senior Fitness Test.
**Lower limb function**	Short Physical Performance Battery [40]	This battery assesses the lower limbs' balance, gait, speed, endurance, and strength. The SPPB measures 3 timed tests: balance in the standing position, walking speed, and chair stands. Each test is scored between 0-4, total score ranges from 0 (poor performance) to 12 (best performance).
**Functional fitness**	Senior Fitness Test [38,39]	Variation of participant's upper/lower body strength, flexibility and aerobic endurance assessed through the different tests from the Senior Fitness Test.
**Functional capacity to perform basic ADLs**	Barthel Index [41]	Measures functional independence on the basic ADLs. The scores vary from 0 to 20; higher scores represent increased functionality and independence.

ADLs, Activities of Daily Living, HADS, Hospital Anxiety and Depression Scale, MEDAS, Mediterranean Diet Adherence Screener Questionnaire, MoCA, Montreal Cognitive Assessment, mRS, Modified Rankin Scale, SPPB, Short Physical Performance Battery

Blinding

Due to the nature of the present intervention, it will not be possible to guarantee a proper blinding to the intervention arm among participants; nevertheless, allocation group will not be actively reported. Regarding the outcomes assessment, interviewers will be independent and blinded to each participants' allocation group.

For peer review only

Data analysis and sample size

The associations between the social and clinical characteristics and feasibility (e.g., recruitment timeframe, adherence, dropouts) and lifestyle outcomes will be quantified through linear or logistic regression models, as applicable. Time in days from the onset of symptoms to complete or partial recovery in each instrumental ADLs will be estimated and illustrated by Kaplan-Meier survival curves. The time comparison between intervention and control groups will be made through the log-rank test. To estimate the association between the intervention and the recovery time, hazard ratios will be estimated, through the computation of univariate and multivariate Cox regression models.

Training of the interviewers and use of standardized procedures for data collection is expected to contribute to a low proportion of missing data, and no imputation is being planned.

To the best of our knowledge, there is no previous information regarding effect sizes and standard deviations regarding the primary outcome related with the recovery of functionality after a TIA or a minor stroke. Nevertheless, a sample size of at least 70 participants (35 per arm) is commonly recommended for a pilot study when estimating the pooled standard deviation.[42] Nevertheless, taking into account the results of a previous RCT evaluating a cardiac model of rehabilitation for reducing risk factors post-TIA and stroke,[43] this sample size will allow to detect mean differences of 5.5 and 6.0 in the changes observed in the Hospital Anxiety and Depression Scale (HADS) - anxiety and depression scores, respectively, between intervention and control groups (assuming a statistical power of 80% and a level of significance of 5%).

To achieve the sample size needed to accomplish the proposed objectives, we expect to invite approximately 100 individuals, since a participation of 70% was previously observed in the

For peer review only

EPIPorto cohort.[44] We also expect a drop-out of 10% from baseline to the first follow-up; nevertheless, and in order to minimize refusals and losses to follow-up, all activities will take place near participants' residence and be scheduled for their convenience.

Contingency plan

This study intents to recruit participants within seven days after the onset of symptoms and start intervention no later than 15 days, which is a considerably short period. This recruitment timeframe may be difficult to attain, so additional measures may have to be adopted, namely receive referrals from other hospitals in neighboring counties (e.g., Porto).

Due to the COVID-19, the number of in-person sessions and participants per group may be reduced. However, if necessary, all activities will be home-based. To minimize the potential impact of the pandemic on participation and retention rates, additional mitigation measures may be adopted, including enlarging the recruitment period or an increase in the sample size.

Patient and public involvement

Patients and public were not involved in the conception, design and dissemination of this study.

For peer review only - http://bmjopen.bmj.com/site/about/guidelines.xhtml

Ethics and Dissemination

Ethics approval was obtained by the Ethics Committee of the ULSM (Ref. Nº 75/CES/JAS) and the Data Protection Officer of the ULSM (Ref. 14/CLPSI/2021) and Institute of Public Health of the University of Porto.

Written informed consent will be obtained from all the participants once the study's objectives and procedures are fully explained. The voluntary nature of participation will be emphasized, clearly stating that they can refuse to participate or leave the study with no penalty associated, namely their right to medical care. There are no expected risks or discomfort other than those arising from the sessions, interviews, anthropometric measures, and the collection of venous blood and 24-hour urinary samples. The intervention group will be covered by personal accident insurance to indemnify any unlikely accident related to the sessions.

To guarantee safety and security of physical exercise, participants with contraindications for exercise practice would not be included in the study. In-person sessions will be preferred, but if sessions are online, some of the exercises will be in a sitting position and participants are encouraged to have someone near them throughout the sessions at home.

This study involves collecting, analysing, and processing sensitive personal data, including contact information, health and clinical data from questionnaires, and participants' clinical records. Therefore, measures are in place to guarantee the confidentiality and anonymity of the participants. All data from clinical records will be collected by clinical members of the research team and privacy will be assured. Health and clinical data will be pseudonymised, with each participant being assigned and identified with a unique numerical code in the study. The principal investigator will be the only person with access to the file containing the correspondence between this unique numerical code and the personally identifiable information. Only specific members of the research team have access to the names and contact

For peer review only - http://bmjopen.bmj.com/site/about/guidelines.xhtml

information of the participants, which will be only used to book evaluations and intervention sessions and resolve any logistical problem throughout the implementation of the intervention program.

Documents with personal identification data and informed consents will be securely stored, separated from other documents and with access limited to the research team, and no personal identifiers will be used in data analyses. The study is in accordance with the Helsinki Declaration of 1964, further amendments,[45] and applicable national and European union legislation.

The implementation of the present study will elucidate the feasibility and impact of a multidomain non-pharmacological program in recovering functionality after a TIA or a minor stroke, which may contribute to delineate further larger RCT and adequate preventive actions. This study will also provide important data to the refinement and validation of the BoT, which will allow a more practical, intensive and economic monitoring of cognitive performance in this specific population. The expected results will be relevant for public health policies, by providing a characterization of residual sequelae of a TIA or a minor stroke, to improve patients' functionality and promote a faster recovery. It may, ultimately, contribute to a better allocation of resources and the creation of synergies between different stakeholders, as well as to improve the management of care.

The findings of this study will be disseminated at several levels, including the publication of scientific papers in international peer-reviewed journals and presentation in relevant national and international conferences. A short training course on secondary prevention post-stroke for public health researchers and students will be also conducted and the project will contribute to the training of researchers through the production of a master dissertation. The main conclusions of the project will be disseminated through press releases and conferences to the media (including newspapers, TV and social media such as Facebook, LinkedIn and Twitter), fostering a connection between science and society.

For peer review only - http://bmjopen.bmj.com/site/about/guidelines.xhtml

Contributors: MG and VTC conceived and designed the study. MG wrote the first version of the manuscript. MJL, ARC, AF, CD collaborated in refining study design and critically revised the manuscript for relevant intellectual content. All authors approved the final manuscript as submitted.

Competing interests: Vítor Tedim Cruz has a shareholder position in Neuroinova, Lda, a start-up company that conceived Brain on Track® and holds registered trademark and commercialization rights.

Funding: This study was funded by national funding from the Foundation for Science and Technology – FCT (Portuguese Ministry of Science, Technology and Higher Education), under the projects UIDB/04750/2020 and LA/P/0064/2020. It was also supported by "Portugal Inovação Social" and co-funded by the Operational Program Social Inclusion and Employment, Portugal 2020 and European Union, through the European Social Fund, under the project "MIND-Matosinhos" (POISE-03-4639-FSE-000793).

Patient and public involvement: Patients and/or the public were not involved in the design, or conduct, or reporting, or dissemination plans of this research.

Patient consent for publication: Not required.

Provenance and peer review: Not commissioned; externally peer reviewed.

For peer review only - http://bmjopen.bmj.com/site/about/guidelines.xhtml

References

1. Easton JD, Saver JL, Albers GW, et al. Definition and evaluation of transient ischemic attack: a scientific statement for healthcare professionals from the American Heart Association/American Stroke Association Stroke Council; Council on Cardiovascular Surgery and Anesthesia; Council on Cardiovascular Radiology and Intervention; Council on Cardiovascular Nursing; and the Interdisciplinary Council on Peripheral Vascular Disease. The American Academy of Neurology affirms the value of this statement as an education tool for neurologists. *Stroke* 2009;**40**:2276–93.
2. Fischer U, Baumgartner A, Arnold M, et al. What is a minor stroke? *Stroke* 2010;**41**:661–6.
3. Béjot Y, Daubail B, Giroud M. Epidemiology of stroke and transient ischemic attacks: current knowledge and perspectives. *Rev Neurol (Paris)* 2016;**172**:59–68.
4. Felgueiras R, Magalhães R, Silva MR, et al. Transient ischemic attack: incidence and early risk of stroke in northern Portugal from 1998–2000 to 2009–2011. *Int J Stroke* 2020;**15**:278–88.
5. Amarenco P, Lavallée PC, Monteiro Tavares L, et al. Five-year risk of stroke after TIA or minor ischemic stroke. *N Engl J Med* 2018;**378**:2182–90.
6. GBD 2019 Stroke Collaborators. Global, regional, and national burden of stroke and its risk factors, 1990-2019: A systematic analysis for the global burden of disease Study 2019. *Lancet Neurol* 2021;**20**:1–26.
7. Dubuc V, Choi P, Hill MD, et al. Can 90-day NIHSS be used for outcome assessment in TIA and minor stroke studies?. *Cerebrovasc Dis* 2015;**40**:97–8.
8. Verbraak M, Hoeksma A, Lindeboom R, et al. Subtle problems in activities of daily living after a transient ischemic attack or an apparently fully recovered non-disabling stroke. *J Stroke Cerebrovasc Dis* 2012;**21**:124–30.
9. Sackley CM, Mant J, McManus RJ, et al. Functional and emotional outcomes after transient ischemic attack: a 12-month prospective controlled cohort study. *Int J Stroke* 2019;**14**:522–9.
10. Carnes-Vendrell A, Deus J, Molina-Seguin J, et al. Depression and apathy after transient Ischemic attack or minor stroke: prevalence, evolution and predictors. *Sci Rep* 2019;**9**.
11. van Rooij FG, Kessels RPC, Richard E, et al. Cognitive impairment in transient ischemic attack patients: a systematic review. *Cerebrovasc Dis* 2016;**42**:1–9.
12. Batchelor F, Williams S, Wijeratne T, et al. Balance and gait impairment in transient ischemic attack and minor stroke. *J Stroke Cerebrovasc Dis* 2015;**24**:2291–7.
13. Koohi N, Vickers DA, Lakshmanan R, et al. Hearing characteristics of stroke patients: prevalence and characteristics of hearing impairment and auditory processing disorders in stroke patients. *J Am Acad Audiol* 2017;**28**:491–505.
14. Turner GM, McMullan C, Atkins L, et al. TIA and minor stroke: a qualitative study of long-term impact and experiences of follow-up care. *BMC Fam Pract* 2019;**20**:176.
15. Fonseca AC, Merwick Á, Dennis M, et al. European Stroke Organisation (ESO) guidelines on management of transient ischaemic attack. *Eur Stroke J* 2021;**6**:CLXIII–CLXXXVI.
16. Cruz VT. MIND-Matosinhos: multiple interventions to prevent cognitive decline. *ClinicalTrials.gov* 2022. https://clinicaltrials.gov/ct2/show/NCT05383443?term=MIND-Matosinhos&draw=2&rank=1 (accessed Jun 2022).
17. Gregório MJ, Rodrigues AM, Salvador C, et al. Validation of the telephone-administered version of the mediterranean diet adherence screener (Medas) questionnaire. *Nutrients* 2020;**12**:1511.
18. Pais-Ribeiro J, Silva I, Ferreira T, et al. Validation study of a portuguese version of the Hospital Anxiety and Depression Scale. *Psychol Health Med* 2007;**12**:225–37.
19. Ginó S, Mendes T, Maroco J, et al. Memory complaints are frequent but qualitatively different in young and elderly healthy people. *Gerontology* 2010;**56**:272–7.
20. Ferreira PL, Antunes P, Ferreira LN, et al. A hybrid modelling approach for eliciting health state preferences: the portuguese EQ-5D-5L value set. *Qual Life Res* 2019;**28**:3163–75.

For peer review only - http://bmjopen.bmj.com/site/about/guidelines.xhtml

21. Ruano L, Branco M, Severo M, et al. Tracking cognitive impairment in multiple sclerosis using the brain on track test: a validation study. *Neurol Sci* 2020;**41**:183–91.
22. Cruz VT, Pais J, Bento V, et al. A rehabilitation tool designed for intensive web-based cognitive training: description and usability study. *JMIR Res Protoc* 2013;**2**:e59.
23. Martins T, Ribeiro JP, Garrett C. [Study of adaptability and validation of the Frenchay Activities Index in a sample of patients with low educational levels affected by stroke]. *Arq Med* 2003;**17**:88–91.
24. Holbrook M, Skilbeck CE. An activities index for use with stroke patients. *Age Ageing* 1983;**12**:166–70.
25. Duro D, Simões MR, Ponciano E, et al. Validation studies of the portuguese experimental version of the Montreal Cognitive Assessment (MoCA): confirmatory factor analysis. *J Neurol* 2010;**257**:728–34.
26. Nasreddine ZS, Phillips NA, Bédirian V, et al. The Montreal Cognitive Assessment, MoCA: a brief screening tool for mild cognitive impairment. *J Am Geriatr Soc* 2005;**53**:695–9.
27. Freitas S, Simões MR, Alves L, et al. Montreal Cognitive Assessment (MoCA): normative study for the portuguese population. *J Clin Exp Neuropsychol* 2011;**33**:989–96.
28. Ruano L, Sousa A, Severo M, et al. Development of a self-administered web-based test for longitudinal cognitive assessment. *Sci Rep* 2016;**6**:19114.
29. Schmand B, Jonker C, Hooijer C, et al. Subjective memory complaints may announce dementia. *Neurology* 1996;**46**:121–5.
30. Herdman M, Gudex C, Lloyd A, et al. Development and preliminary testing of the new five-level version of EQ-5D (EQ-5D-5L). *Qual Life Res* 2011;**20**:1727–36.
31. Wilson JTL, Hareendran A, Grant M, et al. Improving the assessment of outcomes in stroke: use of a structured interview to assign grades on the modified Rankin Scale. *Stroke* 2002;**33**:2243–6.
32. Broderick JP, Adeoye O, Elm J. Evolution of the Modified Rankin Scale and its use in future stroke trials. *Stroke* 2017;**48**:2007–12.
33. Kim D. The effects of hand strength on upper extremity function and activities of daily living in stroke patients, with a focus on right hemiplegia. *J Phys Ther Sci* 2016;**28**:2565–7.
34. Bohannon RW, Peolsson A, Massy-Westropp N, et al. Reference values for adult grip strength measured with a jamar dynamometer: a descriptive meta-analysis. *Physiotherapy* 2006;**92**:11–5.
35. Sousa-Santos AR, Amaral TF. Differences in handgrip strength protocols to identify sarcopenia and frailty - A systematic review. *BMC Geriatr* 2017;**17**:238.
36. Podsiadlo D, Richardson S. The Timed “Up & Go”: A test of basic functional mobility for frail elderly persons. *J Am Geriatr Soc* 1991;**39**:142–8.
37. Springer BA, Marin R, Cyhan T, et al. Normative values for the unipedal stance test with eyes open and closed. *J Geriatr Phys Ther* 2007;**30**:8–15.
38. Langhammer B, Stanghelle JK. Senior fitness test; a useful tool to measure physical fitness in persons with acquired brain injury. *Brain Inj* 2019;**33**:183–8.
39. Langhammer B, Stanghelle JK. The Senior Fitness Test. *J Physiother* 2015;**61**:163.
40. Guralnik JM, Simonsick EM, Ferrucci L, et al. A short physical performance battery assessing lower extremity function: association with self-reported disability and prediction of mortality and nursing home admission. *J Gerontol* 1994;**49**:M85–94.
41. Araújo F, Oliveira A, Pinto C, et al. [Validation of the Barthel Index in a sample of non-institutionalized elderly]. *Revista Portuguesa de Saúde Pública* 2007;**25**:59–66.
42. Teare MD, Dimairo M, Shephard N, et al. Sample size requirements to estimate key design parameters from external pilot randomised controlled trials: a simulation study. *Trials* 2014;**15**.
43. Kirk H, Kersten P, Crawford P, et al. The cardiac model of rehabilitation for reducing cardiovascular risk factors post transient ischaemic attack and stroke: a randomized controlled trial. *Clin Rehabil* 2014;**28**:339–49.

For peer review only

For peer review only - http://bmjopen.bmj.com/site/about/guidelines.xhtml

44. Ramos E, Lopes C, Barros H. Investigating the effect of nonparticipation using a population-based case-control study on myocardial infarction. *Ann Epidemiol* 2004;**14**:437–41.
45. World Medical Association. World Medical Association Declaration of Helsinki: ethical principles for medical research involving human subjects. *JAMA* 2013;**310**:2191–94.

For peer review only

For peer review only - http://bmjopen.bmj.com/site/about/guidelines.xhtml

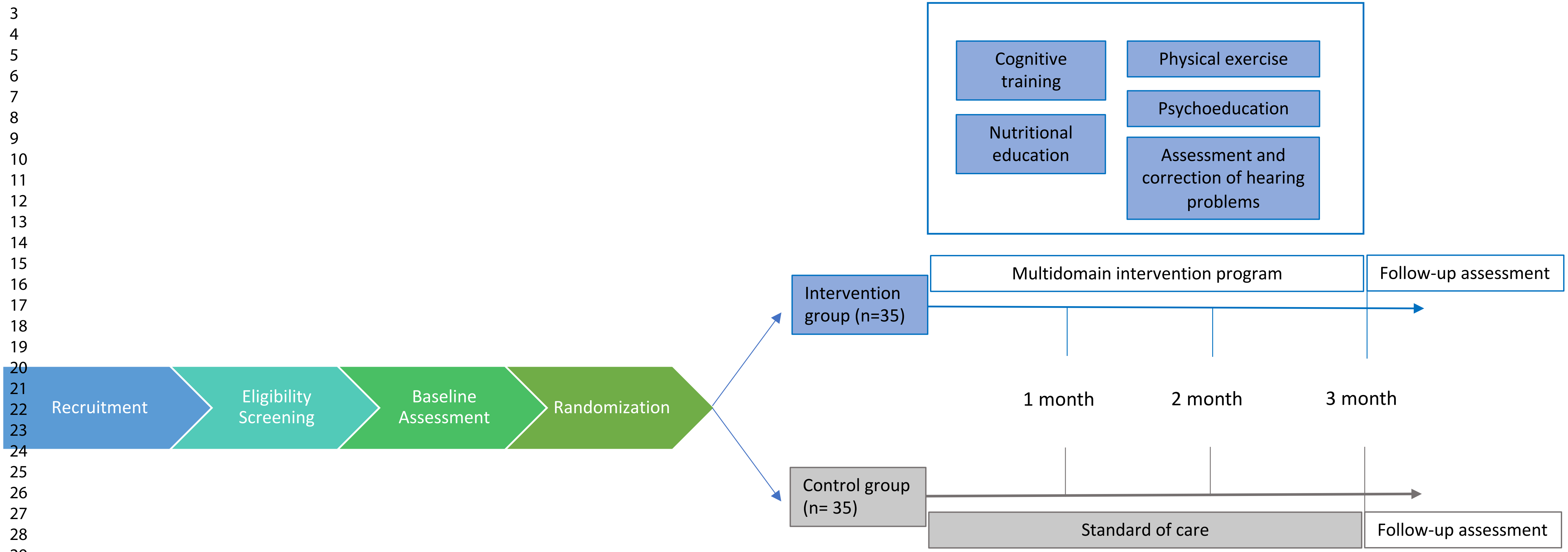
Cognitive training
Physical exercise
Psychoeducation
Nutritional education
Assessment and correction of hearing problems
Multidomain intervention program
Follow-up assessment
Intervention group (n=35)
Recruitment
Eligibility Screening
Baseline Assessment
Randomization
1 month
2 month
3 month
Control group (n= 35)
Standard of care
Follow-up assessment

BMJ Open

A study protocol for a pilot randomised controlled trial evaluating the feasibility and effectiveness of non-pharmacological interventions to recover functionality after a transient ischemic attack or a minor stroke: the "Back to Normal" trial

Journal:	*BMJ Open*
Manuscript ID	bmjopen-2022-069593.R1
Article Type:	Protocol
Date Submitted by the Author:	07-Mar-2023
Complete List of Authors:	Gonçalves, Micaela; University of Porto Epidemiology Research Unit<br>Lima , Maria João; Unidade Local de Saúde de Matosinhos EPE, Neurology<br>Fonseca, Ângelo ; Unidade Local de Saúde de Matosinhos EPE, Neurology<br>Duque, Cristina; Unidade Local de Saúde de Matosinhos EPE, Neurology<br>Costa, Ana ; University of Porto Epidemiology Research Unit; FMUP, Departamento de Ciências da Saúde Pública e Forenses e Educação Médica<br>Cruz, Vitor; University of Porto Epidemiology Research Unit; Unidade Local de Saúde de Matosinhos EPE, Neurology
**Primary Subject Heading**:	Neurology
Secondary Subject Heading:	Public health
Keywords:	Stroke < NEUROLOGY, PUBLIC HEALTH, PREVENTIVE MEDICINE

SCHOLARONE™ Manuscripts

A study protocol for a pilot randomised controlled trial evaluating the feasibility and effectiveness of non-pharmacological interventions to recover functionality after a transient ischemic attack or a minor stroke: the "Back to Normal" trial

Micaela Gonçalves[a,b], Maria João Lima[c], Ângelo Fonseca[c], Cristina Duque[c], Ana Rute Costa[a,b,d], Vítor Tedim Cruz[a,b,c]

[a] EPIUnit – Instituto de Saúde Pública, Universidade do Porto, Rua das Taipas, n° 135, 4050-600 Porto, Portugal

[b] Laboratório para a Investigação Integrativa e Translacional em Saúde Populacional (ITR), Universidade do Porto, Rua das Taipas, n° 135, 4050-600 Porto, Portugal

[c] Serviço de Neurologia, Hospital Pedro Hispano, Unidade Local de Saúde de Matosinhos, Rua Dr. Eduardo Torres, 4464-513 Senhora da Hora, Portugal.

[d] Departamento de Ciências da Saúde Pública e Forenses e Educação Médica, Faculdade de Medicina da Universidade do Porto, Alameda Professor Hernâni Monteiro, 4200-319 Porto, Portugal.

Corresponding author:

Micaela Gonçalves

Instituto de Saúde Pública da Universidade do Porto (ISPUP)

Rua das Taipas, nº 135

4050-600 Porto, Portugal

Telephone: +351 222061820

E-mail: micaela.goncalves@ispup.up.pt

For peer review only

Abstract

Introduction: Transient ischemic attack (TIA) and minor stroke are frequently assumed as temporary or non-disabling events. However, evidence suggests that these patients can experience relevant impairment and functional disability. Therefore, the present study aims to evaluate the feasibility and effectiveness of a three-month multidomain intervention program, composed of five non-pharmacological strategies, aimed at accelerating return to pre-event level of functionality in patients with TIA or minor stroke.

Methods and Analysis: Patients diagnosed with a TIA or a minor stroke are being recruited at the emergency or neurology departments of the Hospital Pedro Hispano, located in Matosinhos, Portugal (n=70). Those who accept to participate will be randomly allocated to two groups (1:1): (a) Intervention – receives a three-months combined approach, initiating early post-event, composed of cognitive training, physical exercise, nutrition, psychoeducation and assessment/correction of hearing loss; (b) Control – participants will not be subject to any intervention. Both groups will receive the usual standard of care provided to these diseases. Recruitment began in May 2022 and is expected to continue until March 2023. Sociodemographic characteristics, lifestyles, health status, cognitive function, symptoms of anxiety and depression, and quality of life will be assessed; as well as anthropometry, blood pressure and physical condition. Time to complete or partial recovery of instrumental activities of daily living will be assessed using an adapted version of the Frenchay Activities Index. All participants will be evaluated before the intervention and after 3 months.

Ethics and Dissemination: This study was approved by the Ethics Committee of the Local Health Unit of Matosinhos (Ref. 75/CES/JAS). Written informed consent will be required from all the participants; data protection and confidentiality will be also ensured. The findings of this project

For peer review only - http://bmjopen.bmj.com/site/about/guidelines.xhtml

are expected to be submitted for publication in scientific articles, and the main results will be presented at relevant scientific meetings.

Trial Registration Number: NCT05369637

Keywords: Ischemic Attack, Transient; Minor Stroke; Non-Pharmacological Interventions; Activities of Daily Living; Randomised Controlled Trial

Word Count: 3592

For peer review only

For peer review only - http://bmjopen.bmj.com/site/about/guidelines.xhtml

Strengths and Limitations of this study

- This protocol describes a pilot randomized controlled trial addressing the effect of an innovative multidomain non-pharmacological intervention program on post-transient ischemic attack and minor stroke functionality.
- The intervention program will start early after the diagnosis of the vascular event, maximizing health gain and targeting brain plasticity.
- The main outcome is based on the time to recover instrumental activities of daily living after the event, which is more suitable due to the characteristics of residual impairment.
- This study will explore a wide range of different outcomes, including study feasibility, cognitive function, lifestyle behaviors, physical and emotional status.
- Given the characteristics of the intervention, blinding of the participants is not possible. In addition, the main outcome is evaluated by self-report and is based on a pre-defined list of activities of daily living. Therefore, we cannot exclude the existence of recall bias regarding our main outcome.

For peer review only

Introduction

A Transient Ischemic Attack (TIA) is defined as a "transient episode of neurological dysfunction caused by focal brain, spinal cord, or retinal ischemia, without acute infarction" by the American Heart Association,[1] while a minor stroke is suggested to occur when a patient has a score of ≤3 in the National Institutes of Health Stroke Scale (NIHSS).[2] Population-based studies have shown that the age-adjusted incidence of TIA in the European population ranges from 28 to 59/100 000/year[3] and, in Portugal, a higher annual TIA incidence of 74/100 00/ people was estimated between 2009–2011.[4] This is particularly relevant since previous studies have described an increased risk of stroke after these events,[5] and, in 2019, stroke was the third leading cause of disability-adjusted life years (DALYs).[6]

The study of the overall morbidity associated with a TIA or a minor stroke is often neglected, since most of the time they are assumed as being transient or non-disabling events. However, evidence suggests that these patients can experience relevant disability even with a NIHSS of 0 or 1.[7] A decreased performance in activities of daily living (ADLs) up to 1-year post-event was also observed, including in patients without any apparent physical disability.[8,9] Other studies showed a high prevalence of depression and apathy,[10] cognitive impairment,[11] physical deficits[12] and hearing degeneration,[13] with implications on patients' return to work, social relations and activities.[14] Moreover, European guidelines on management of TIA recommend for early initiation of secondary prevention following such an event,[15] focusing particularly on the leading risk factors for stroke, namely high systolic blood pressure, body mass index, fasting plasma glucose and smoking.[6]

Taken together, these findings highlight the importance of multidomain interventions aiming to promote functioning levels among patients with TIA or minor stroke, and to develop preventive

For peer review only - http://bmjopen.bmj.com/site/about/guidelines.xhtml

actions and health promotion plans specifically designed for this group. As such, this pilot study primarily aims to explore the feasibility and the effectiveness of an innovative multidomain intervention program, composed of five strategies: cognitive training, physical exercise, nutrition education, psychoeducation and assessment/correction of hearing problems. These are aimed at accelerating the return to pre-event functionality in patients with a TIA or a minor stroke diagnosis. The primary objectives are as follows:

1) To assess the feasibility of methodology for early recruitment, collecting information and delivering the interventions;

2) To estimate the effectiveness of this program on the time to recovery in each instrumental ADLs.

As secondary objectives, the effect of this intervention on cognitive function, adherence to the Mediterranean diet, physical function, basic ADLs, symptoms of anxiety and depression, quality of life, anthropometric measures and blood pressure, will be also assessed.

For peer review only

Methods and Analysis

Study design

The present study is a two-arm parallel, superiority, pilot randomised controlled trial (RCT), which will be nested in the ongoing project "Multiple Interventions to Prevent Cognitive Decline" (MIND-Matosinhos).[16] This proposed pilot study will provide crucial information about the logistic feasibility, provide estimates of eligibility, recruitment and attrition rates, optimizing the study design. It will also enable a greater precision in the power and sample size calculations of a future full-scale study.

The primary endpoints will be the feasibility of this multidomain program, as well as the time to recovery to pre-event functionality after the occurrence of a TIA or a minor stroke. A block randomisation with a 1:1 allocation will be performed.

Setting

This pilot RCT will be conducted at the department of neurology of the Hospital Pedro Hispano E.P.E (HPH), as part of the Local Health Unit of Matosinhos (ULSM), which is integrated in the Portuguese National Health Service. The HPH is located at the Matosinhos municipality, Northern Portugal, and is responsible for providing care to 318 000 inhabitants from the municipalities of Matosinhos, Vila do Conde and Póvoa de Varzim.

Eligibility criteria

Patients aged 18-85 years old and with a clinical diagnosis of TIA or minor stroke as defined by a National Institute of Health Stroke Scale (NIHSS) score ≤3 are eligible for this study. Additional inclusion criteria include: the onset of symptoms within the last seven days; first-time stroke or TIA; at least four or more years of education; discharged home without the need for inpatient

For peer review only - http://bmjopen.bmj.com/site/about/guidelines.xhtml

rehabilitation; modified Rankin Scale (mRS) 0 to 2. Patients will be excluded if they are unable to attend the face-to-face intervention sessions, or if they have: previous diagnosis of dementia or severe disability; contraindication for physical exercise, severe loss of hearing, vision, or communication skills; frailty, reduced life expectancy due to severe disease or need for regular treatments that compete with availability for intervention (e.g., chemotherapy, haemodialysis).

Recruitment

Participants will be enrolled through consecutive sampling from the Neurology Department (Stroke or Emergency care units) of HPH. Healthcare professionals from the recruitment site will be informed about the protocol and their potential role, and an experienced researcher will be on-site to provide coordination. The Neurology medical team will be responsible for screening eligibility and obtaining written informed consent for all the participants. Recruitment started on May 11, 2022.

Randomization

The participants will be randomly assigned (1:1) to the intervention or control group after the baseline assessment. Randomization will be done using a computer-generated program, using random blocks of four sizes. A researcher who is not immediately engaged in the project will implement the randomization process.

For peer review only

Data collection

Participants will be evaluated at baseline (before the beginning of the intervention program) and at three months, as depicted in Figure 1. Data on sociodemographic (birth date, sex, address, marital status, education, occupation, income), medical history, current medication, lifestyle (smoking and alcohol use, physical activity and sedentary behaviors) and adherence to Mediterranean diet [17] will be collected through structured questionnaires applied by a trained researcher. Anxiety and depression,[18] memory complaints [19] and quality of life [20] will be evaluated through self-administered questionnaires validated for the Portuguese population. Additionally, long term cognition data will be also collected every 3 months for a period of up to 10 years using Brain on Track® (BoT), a web-based tool for remote longitudinal assessment of different cognitive domains.[21] The outcome measure section describes the instruments in detail.

Data regarding clinical diagnosis, onset of symptoms, hospital admission and discharge date, mRS score, NIHSS score, medical history of cerebrovascular events, age, and need for inpatient/outpatient rehabilitation will be retrieved from the patient´s medical record. Clinical characteristics of the cerebrovascular event will also be collected.

Intervention

The intervention will be implemented over three months and will comprise five different components, including cognitive training, physical exercise, nutritional education, psychoeducation and assessment and correction of hearing problems. Only the intervention group will be exposed to all or any of the components of this comprehensive intervention program. Both groups will continue to receive the usual standard of care provided to this type of clinical disease in the hospital setting. The usual standard of care consists of the initiation of

For peer review only - http://bmjopen.bmj.com/site/about/guidelines.xhtml

the appropriate secondary prevention and referral to the outpatient cerebrovascular diseases clinic (telephone call at 1-month post-event and in-person appointment at 3-months post-event). Depending on the clinical presentation and neurological deficits, patients can also be referred to an outpatient physical medicine and rehabilitation appointment.

Since early intervention post-vascular event is decisive, given the time interval between neurological injury and the onset of nervous function recovery activities, intervention sessions are aimed to begin within no later than 15 days after the cerebrovascular event. Considering this timeline, once the participants are recruited and randomized, the intervention will start immediately, especially cognitive training.

To minimize refusals and losses to follow-up, all evaluations and sessions will be scheduled by the participant's preference. A written plan with the intervention schedules will be given and sessions reminded through email or phone messages in the day before (day, time, and location). If one intervention session is missed, efforts will be placed to arrange another suitable time in another group, and individualized meetings will be rescheduled. If not possible, the missed appointment will be documented, and the dropout or non-compliance reasons will be registered for further analysis.

<u>Cognitive training</u>

Monthly in-person sessions in groups of a minimum of five participants (60 min) will be conducted by a psychologist. Additionally, during the three months a daily home-based cognitive training individual (30 minutes) sessions using the COGWEB® [22] online platform will be performed. This online computer tool provides cognitive training tailored to each patient's deficits with different difficulty levels, allowing continuous treatment monitoring, clinical evolution, and adherence compliance.[22] Exercises are aimed at different cognitive domains, such as attention, memory, executive function, language and calculus.[22] Participants have

For peer review only

access to instructions for each exercise before initiating the session, displayed on the screen and through the speakers. Instructions can be seen at any moment, even throughout the session. Participants without access to a computer will receive paper/pencil equivalent home-based cognitive exercises.

Physical exercise

Physical exercise sessions in-person or remotely twice a week with a duration of up to 60 minutes, guided and supervised by a graduated physical exercise technician, will be conducted. Activities will be tailored to each participant tolerance, medical history, and comorbidities, focusing on impaired balance and weakness. During exercise sessions, the exercise technician will track symptoms of chest pain, shortness of breath, wheezing, dyspnoea, light-headedness, nausea, among others. Small groups allow to set short and attainable goals with the participants and have a graded exercise program, starting with reduced intensity and gradually increasing.

In-person sessions will be preferred, however if online sessions are needed, the participants without a computer will receive booklets with physical exercise instructions twice a week and the researcher will contact the participants to assess adherence to the exercises.

Nutritional education

Intervention arm will include individual appointments (three appointments with a nutritionist, once a month with a duration of 45 min to 60min) and in-person group cooking sessions (once a month with a duration of 90 min). During individual sessions, the nutritionist will emphasize the benefits of adhering to the Mediterranean diet and salt reduction, particularly for overall cardiovascular health advantages. Group sessions start with the nutritionist's presentation of healthy recipes and further analysis of nutritional values, then the participants will undergo its preparation. In these sessions, participants can bring their partners or one family member.

For peer review only - http://bmjopen.bmj.com/site/about/guidelines.xhtml

Psychoeducation

Monthly in-person psychoeducation sessions (60 minutes) in groups of a minimum of five participants will be conducted by a psychologist. These sessions will support people in understanding, exploring, and self-managing their emotions and impairments, through peer support, and promotion of lifestyle changes.

Assessment and correction of hearing problems

At the beginning of the intervention program, a hearing assessment and correction will be performed. This evaluation will be conducted by otolaryngologists and audiologists, who will evaluate previous hearing problems and use of hearing aids, and includes otoscopy and audiogram. Those detected with any hearing problem will be referred for a medical appointment with an otolaryngologist.

Primary outcome measures

The primary outcomes for the study are related with its feasibility and time to partial or complete recovery of each instrumental ADLs.

To evaluate the feasibility of this study, the following primary outcomes will be assessed:

- Recruitment timeframe: proportion of participants recruited within seven days after onset of symptoms. It will be assessed by dividing the number of participants recruited within the time frame divided by the total number of participants.
- Beginning of the intervention: proportion of participants that started any of the interventions no later than 15 days after onset of symptoms.

For peer review only

- Adherence to each component of the intervention: proportion of adherence to each component of the intervention and different intervention modalities (remote/in person), calculated as the number of sessions attended divided by the total number of sessions provided. For the COGWEB® training, the outcome will be the absolute number of sessions.
- Dropout: proportion of participants who dropped out of the study, calculated as the number of participants who dropped out after attending at least one session divided by the total number of participants who attended at least one session.

Time to recovery in each instrumental activity of daily living will be measured by an adapted version of the Frenchay Activities Index (FAI). The translated and validated version of the FAI – adapted version for the Portuguese population [23] will be used to assess the instrumental ADLs functional performance at baseline and follow-up at three months. The version used in this study is based on the perception of the patient's capacity to perform a specific instrumental ADLs instead of its frequency, as stated in the original scale developed by Holbrook and Skilbeck.[24] The scale consists of 15 activities, scored from 1 to 4 (1-Could not do at all; 2-very difficult; 3-a little difficult; 4-not difficult at all).[23] Although the original index suggests that total scores for men and women should be considered separately,[24] in the present study the score of each activity will be analysed independently, as the interest in exploring the impact of the programme in each activity.

For the present study, only activities that are part of participants' daily lives will be considered, namely those that were performed at least once in the last 12 months. Time to recovery will be defined as the time from the start of the intervention program to the time of complete or partial recovery. Each participant will report their level of performance in each activity at the baseline and follow-up at three months. In the baseline, if a participant scores a specific activity as altered (score 1 to 3), their level of performance before the TIA or minor stroke will be confirmed to

For peer review only - http://bmjopen.bmj.com/site/about/guidelines.xhtml

understand if the change in the activity performance is related with the cerebrovascular event. It will be also inquired and recorded the date when a difficulty in a certain activity ceased. The average of activities altered post-event (score 1,2,3) at baseline and the average of activities that improved in both groups upon completing the intervention program will be measured.

Secondary outcome measures

Secondary outcomes and the instruments that will be used for their assessments are described in Table 1 and Table 2. Briefly, secondary endpoints related with the study's feasibility will be measured, namely time of follow-up, implemented sessions and complete assessment of participants (Table 1). Additionally, cognitive function, quality of life, symptoms of anxiety and depression, as well as functional capacity to perform basic ADLs will be evaluated through questionnaires validated for the Portuguese population (Table 2). Physical condition, blood pressure, and anthropometrics will be ascertained by trained interviewers. Levels of glycated haemoglobin, and 24-hour urinary excretion of sodium and potassium will be also measured. The secondary outcomes described on the Table 2 will be also measured at baseline and follow-up at three months.

Table 1. Description of the secondary feasibility outcomes.

OUTCOME	DESCRIPTION
**Time of follow-up**	Calculated by the number of days between the participant's first and last session attended.
**Implemented sessions**	Proportion of sessions implemented, calculated as the number of sessions that the research team was able to implement divided by the total number of sessions planned.
**Complete assessment of participants**	Proportion of participants with full information at baseline and follow-up points, calculated by dividing the number of participants with complete information by the number of participants evaluated.

For peer review only - http://bmjopen.bmj.com/site/about/guidelines.xhtml

Table 2. Description of the instruments used for the evaluation of secondary outcomes.

OUTCOME	INSTRUMENT	DESCRIPTION
**Cognitive performance**	MoCA [25–27]	Brief screening tool to detect mild cognitive impairment. It assesses eight cognitive domains: visuospatial ability, executive function, attention, concentration, working memory, language, verbal memory, and orientation, with scores from 0 to 30. Higher scores represent better cognitive performance, and one point is added for participants with less than 12 years of education.
	Brain on Track® [21,28]	Self-administered computerized cognitive test for remote longitudinal assessment. It evaluates different cognitive domains through exercises, such as attention, memory, executive functions, language, calculation, constructive capacity, and visuospatial processing. The score range is the maximum number of correct answers within a limit of time. Higher scores are representative of better cognitive performance.
**Memory Complaints**	Subjective Memory Complaints Scale [19,29]	This scale comprises 10 questions regarding subjective complaints of memory. This scale varies from 0 (best score) to 21 points (worst score).
**Adherence to the Mediterranean diet**	MEDAS questionnaire [17]	The MEDAS questionnaire is composed by 14 questions associated with eating habits and frequency of food consumption. The score ranges between 0 (lowest adherence to the Mediterranean diet) to 14 points (highest adherence to the Mediterranean diet). A score greater than 9 points demonstrates a good adherence to the Mediterranean diet.
**Anxiety and Depression**	HADS [18]	Scale with 14 questions, subdivided in two subscales: anxiety (HADS-A) and depression (HADS-D). This scale varies from 0 (best score) to 21 (worst score) for each sub-scale. The optimal cut-off points differ significantly in the literature. For this study, a score higher than or equal to 11 represents a case of anxiety or depression, as applicable.
**Reported quality of life**	EQ-5D-5L [20,30]	This scale is subdivided into two subscales: a) five multiple-choice questions, with five response options, which results in a score that fluctuates from 5 (best score) to 25 points (worst score). One digit is provided for each dimension, combining in a 5-digit code. A total of 3125 possible health states are delineated to describe the patient's state of health. b) vertical visual analogic scale, which varies from 0 (worst score) to 100 (best score).
**Disability**	Modified Rankin Scale [31,32]	Simple scale that assesses functional capacity, referring to limitations in "activity and changes in lifestyle". The scores run from 0 (perfect health) to 6 (dead). It will be considered disability as an mRS of > 1 and a one-point change on the scale will be clinically significant.
**Handgrip strength**	Dynamometer [33–35]	Force applied in each hand in kilograms using the JAMAR dynamometer. The handgrip can be a relevant predictor of the physical function of the upper body and influences the capacity to perform ADLs. Participant will be in a seated position with the elbow flexed at 90° degrees. Three alternating hand sequential measurements will be performed and registered. It is relevant to document the dominant hand and the affected and non-affected hands if the case.
**Agility**	Time UP and Go test scale [36]	It consists of counting the participant's time to complete the following routine: standing up from a chair, walking for 3 meters (following the marks on the floor), returning to the chair, and sitting down as fast as possible. It provides important information about functional capacity, gait, dynamic balance, and speed. The time will be counted from when the participant's back leaves the chair until sitting again with their back supported. It will be considered a cut-off level of ≤ 10 seconds to identify normal mobility.

For peer review only - http://bmjopen.bmj.com/site/about/guidelines.xhtml

	Unipedal stance test [37]	This test is performed in time units (seconds) and the score varies from 0 (worse score) to 45 seconds (best score). The researcher will use a stopwatch and count the amount of time the participant can stand on one leg, of their choice, with their eyes open.
	Senior Fitness Test [38,39]	Variation of participant's agility and dynamic balance assessed using the 8-foot distance test (2,44 meters) from the Senior Fitness Test.
**Lower limb function**	Short Physical Performance Battery [40]	This battery assesses the lower limbs' balance, gait, speed, endurance, and strength. The SPPB measures 3 timed tests: balance in the standing position, walking speed, and chair stands. Each test is scored between 0-4, total score ranges from 0 (poor performance) to 12 (best performance).
**Functional fitness**	Senior Fitness Test [38,39]	Variation of participant's upper/lower body strength, flexibility and aerobic endurance assessed through the different tests from the Senior Fitness Test.
**Functional capacity to perform basic ADLs**	Barthel Index [41]	Measures functional independence on the basic ADLs. The scores vary from 0 to 20; higher scores represent increased functionality and independence.

ADLs, Activities of Daily Living, HADS, Hospital Anxiety and Depression Scale, MEDAS, Mediterranean Diet Adherence Screener Questionnaire, MoCA, Montreal Cognitive Assessment, mRS, Modified Rankin Scale, SPPB, Short Physical Performance Battery

Blinding

Due to the nature of the present intervention, it will not be possible to guarantee a proper blinding to the intervention arm among participants; nevertheless, allocation group will not be actively reported. Regarding the outcomes assessment, interviewers will be independent and blinded to each participants' allocation group.

For peer review only

For peer review only - http://bmjopen.bmj.com/site/about/guidelines.xhtml

Data analysis and sample size

The associations between the social and clinical characteristics and feasibility (e.g., recruitment timeframe, adherence, dropouts) and lifestyle outcomes will be quantified through linear or logistic regression models, as applicable. Time in days from the onset of symptoms to complete or partial recovery in each instrumental ADLs will be estimated and illustrated by Kaplan-Meier survival curves. The time comparison between intervention and control groups will be made through the log-rank test. To estimate the association between the intervention and the recovery time, hazard ratios will be estimated, through the computation of univariate and multivariate Cox regression models.

Training of the interviewers and use of standardized procedures for data collection is expected to contribute to a low proportion of missing data, and no imputation is being planned.

To the best of our knowledge, there is no previous information regarding effect sizes and standard deviations regarding the primary outcome related with the recovery of functionality after a TIA or a minor stroke. Nevertheless, a sample size of at least 70 participants (35 per arm) is commonly recommended for a pilot study when estimating the pooled standard deviation.[42] Nevertheless, taking into account the results of a previous RCT evaluating a cardiac model of rehabilitation for reducing risk factors post-TIA and stroke,[43] this sample size will allow to detect mean differences of 5.5 and 6.0 in the changes observed in the Hospital Anxiety and Depression Scale (HADS) - anxiety and depression scores, respectively, between intervention and control groups (assuming a statistical power of 80% and a level of significance of 5%).

To achieve the sample size needed to accomplish the proposed objectives, we expect to invite approximately 100 individuals, since a participation of 70% was previously observed in the

For peer review only - http://bmjopen.bmj.com/site/about/guidelines.xhtml

EPIPorto cohort.[44] We also expect a drop-out of 10% from baseline to the first follow-up; nevertheless, and in order to minimize refusals and losses to follow-up, all activities will take place near participants' residence and be scheduled for their convenience.

Contingency plan

This study intents to recruit participants within seven days after the onset of symptoms and start intervention no later than 15 days, which is a considerably short period. This recruitment timeframe may be difficult to attain, so additional measures may have to be adopted, namely receive referrals from other hospitals in neighboring counties (e.g., Porto).

Due to the COVID-19, the number of in-person sessions and participants per group may be reduced. However, if necessary, all activities will be home-based. To minimize the potential impact of the pandemic on participation and retention rates, additional mitigation measures may be adopted, including enlarging the recruitment period or an increase in the sample size.

Patient and public involvement

Patients and public were not involved in the conception, design and dissemination of this study.

For peer review only - http://bmjopen.bmj.com/site/about/guidelines.xhtml

Ethics and Dissemination

Ethics approval was obtained by the Ethics Committee of the ULSM (Ref. Nº 75/CES/JAS) and the Data Protection Officer of the ULSM (Ref. 14/CLPSI/2021) and Institute of Public Health of the University of Porto. Any substantive protocol amendment will be reported to the respective Ethics Committee for approval.

Written informed consent will be obtained from all the participants once the study's objectives and procedures are fully explained. The voluntary nature of participation will be emphasized, clearly stating that they can refuse to participate or leave the study with no penalty associated, namely their right to medical care. There are no expected risks or discomfort other than those arising from the sessions, interviews, anthropometric measures, and the collection of venous blood and 24-hour urinary samples. The intervention group will be covered by personal accident insurance to indemnify any unlikely accident related to the sessions. Due to the expected low risks and the study small sample size, a data monitoring committee was not established.

To guarantee safety and security of physical exercise, participants with contraindications for exercise practice would not be included in the study. In-person sessions will be preferred, but if sessions are online, some of the exercises will be in a sitting position and participants are encouraged to have someone near them throughout the sessions at home.

This study involves collecting, analysing, and processing sensitive personal data, including contact information, health and clinical data from questionnaires, and participants' clinical records. Therefore, measures are in place to guarantee the confidentiality and anonymity of the participants. All data from clinical records will be collected by clinical members of the research team and privacy will be assured. Health and clinical data will be pseudonymised, with each participant being assigned and identified with a unique numerical code in the study. The principal investigator will be the only person with access to the file containing the

For peer review only

correspondence between this unique numerical code and the personally identifiable information. Only specific members of the research team have access to the names and contact information of the participants, which will be only used to book evaluations and intervention sessions and resolve any logistical problem throughout the implementation of the intervention program.

Documents with personal identification data and informed consents will be securely stored, separated from other documents and with access limited to the research team, and no personal identifiers will be used in data analyses. Only the research team will have access to the study data set, saved on a password-protected secure computer. The study is in accordance with the Helsinki Declaration of 1964, further amendments,[45] and applicable national and European union legislation.

The implementation of the present study will elucidate the feasibility and impact of a multidomain non-pharmacological program in recovering functionality after a TIA or a minor stroke, which may contribute to delineate further larger RCT and adequate preventive actions. This study will also provide important data to the refinement and validation of the BoT, which will allow a more practical, intensive and economic monitoring of cognitive performance in this specific population. The expected results will be relevant for public health policies, by providing a characterization of residual sequelae of a TIA or a minor stroke, to improve patients' functionality and promote a faster recovery. It may, ultimately, contribute to a better allocation of resources and the creation of synergies between different stakeholders, as well as to improve the management of care.

The findings of this study will be disseminated at several levels, including the publication of scientific papers in international peer-reviewed journals and presentation in relevant national and international conferences. A short training course on secondary prevention post-stroke for public health researchers and students will be also conducted and the project will contribute to

For peer review only - http://bmjopen.bmj.com/site/about/guidelines.xhtml

the training of researchers through the production of a master dissertation. The main conclusions of the project will be disseminated through press releases and conferences to the media (including newspapers, TV and social media such as Facebook, LinkedIn and Twitter), fostering a connection between science and society.

For peer review only

For peer review only - http://bmjopen.bmj.com/site/about/guidelines.xhtml

Contributors: MG and VTC conceived and designed the study. MG wrote the first version of the manuscript. MJL, ARC, AF, CD collaborated in refining study design and critically revised the manuscript for relevant intellectual content. All authors approved the final manuscript as submitted.

Competing interests: Vítor Tedim Cruz has a shareholder position in Neuroinova, Lda, a start-up company that conceived Brain on Track® and holds registered trademark and commercialization rights.

Funding: This study was funded by national funding from the Foundation for Science and Technology – FCT (Portuguese Ministry of Science, Technology and Higher Education), under the projects UIDB/04750/2020 and LA/P/0064/2020. It was also supported by "Portugal Inovação Social" and co-funded by the Operational Program Social Inclusion and Employment, Portugal 2020 and European Union, through the European Social Fund, under the project "MIND-Matosinhos" (POISE-03-4639-FSE-000793). Funders will not have any influence on the study design, execution, interpretation of data, writing and publication of manuscripts.

Patient consent for publication: Not required.

Provenance and peer review: Not commissioned; externally peer reviewed.

For peer review only

References

1. Easton JD, Saver JL, Albers GW, et al. Definition and evaluation of transient ischemic attack: a scientific statement for healthcare professionals from the American Heart Association/American Stroke Association Stroke Council; Council on Cardiovascular Surgery and Anesthesia; Council on Cardiovascular Radiology and Intervention; Council on Cardiovascular Nursing; and the Interdisciplinary Council on Peripheral Vascular Disease. The American Academy of Neurology affirms the value of this statement as an education tool for neurologists. *Stroke* 2009;**40**:2276–93.
2. Fischer U, Baumgartner A, Arnold M, et al. What is a minor stroke? *Stroke* 2010;**41**:661–6.
3. Béjot Y, Daubail B, Giroud M. Epidemiology of stroke and transient ischemic attacks: current knowledge and perspectives. *Rev Neurol (Paris)* 2016;**172**:59–68.
4. Felgueiras R, Magalhães R, Silva MR, et al. Transient ischemic attack: incidence and early risk of stroke in northern Portugal from 1998–2000 to 2009–2011. *Int J Stroke* 2020;**15**:278–88.
5. Amarenco P, Lavallée PC, Monteiro Tavares L, et al. Five-year risk of stroke after TIA or minor ischemic stroke. *N Engl J Med* 2018;**378**:2182–90.
6. GBD 2019 Stroke Collaborators. Global, regional, and national burden of stroke and its risk factors, 1990-2019: A systematic analysis for the global burden of disease Study 2019. *Lancet Neurol* 2021;**20**:1–26.
7. Dubuc V, Choi P, Hill MD, et al. Can 90-day NIHSS be used for outcome assessment in TIA and minor stroke studies?. *Cerebrovasc Dis* 2015;**40**:97–8.
8. Verbraak M, Hoeksma A, Lindeboom R, et al. Subtle problems in activities of daily living after a transient ischemic attack or an apparently fully recovered non-disabling stroke. *J Stroke Cerebrovasc Dis* 2012;**21**:124–30.
9. Sackley CM, Mant J, McManus RJ, et al. Functional and emotional outcomes after transient ischemic attack: a 12-month prospective controlled cohort study. *Int J Stroke* 2019;**14**:522–9.
10. Carnes-Vendrell A, Deus J, Molina-Seguin J, et al. Depression and apathy after transient Ischemic attack or minor stroke: prevalence, evolution and predictors. *Sci Rep* 2019;**9**.
11. van Rooij FG, Kessels RPC, Richard E, et al. Cognitive impairment in transient ischemic attack patients: a systematic review. *Cerebrovasc Dis* 2016;**42**:1–9.
12. Batchelor F, Williams S, Wijeratne T, et al. Balance and gait impairment in transient ischemic attack and minor stroke. *J Stroke Cerebrovasc Dis* 2015;**24**:2291–7.
13. Koohi N, Vickers DA, Lakshmanan R, et al. Hearing characteristics of stroke patients: prevalence and characteristics of hearing impairment and auditory processing disorders in stroke patients. *J Am Acad Audiol* 2017;**28**:491–505.
14. Turner GM, McMullan C, Atkins L, et al. TIA and minor stroke: a qualitative study of long-term impact and experiences of follow-up care. *BMC Fam Pract* 2019;**20**:176.
15. Fonseca AC, Merwick Á, Dennis M, et al. European Stroke Organisation (ESO) guidelines on management of transient ischaemic attack. *Eur Stroke J* 2021;**6**:CLXIII–CLXXXVI.
16. Cruz VT. MIND-Matosinhos: multiple interventions to prevent cognitive decline. *ClinicalTrials.gov* 2022. https://clinicaltrials.gov/ct2/show/NCT05383443?term=MIND-Matosinhos&draw=2&rank=1 (accessed Jun 2022).
17. Gregório MJ, Rodrigues AM, Salvador C, et al. Validation of the telephone-administered version of the mediterranean diet adherence screener (Medas) questionnaire. *Nutrients* 2020;**12**:1511.
18. Pais-Ribeiro J, Silva I, Ferreira T, et al. Validation study of a portuguese version of the Hospital Anxiety and Depression Scale. *Psychol Health Med* 2007;**12**:225–37.
19. Ginó S, Mendes T, Maroco J, et al. Memory complaints are frequent but qualitatively different in young and elderly healthy people. *Gerontology* 2010;**56**:272–7.
20. Ferreira PL, Antunes P, Ferreira LN, et al. A hybrid modelling approach for eliciting health state preferences: the portuguese EQ-5D-5L value set. *Qual Life Res* 2019;**28**:3163–75.

For peer review only

21. Ruano L, Branco M, Severo M, et al. Tracking cognitive impairment in multiple sclerosis using the brain on track test: a validation study. *Neurol Sci* 2020;**41**:183–91.
22. Cruz VT, Pais J, Bento V, et al. A rehabilitation tool designed for intensive web-based cognitive training: description and usability study. *JMIR Res Protoc* 2013;**2**:e59.
23. Martins T, Ribeiro JP, Garrett C. [Study of adaptability and validation of the Frenchay Activities Index in a sample of patients with low educational levels affected by stroke]. *Arq Med* 2003;**17**:88–91.
24. Holbrook M, Skilbeck CE. An activities index for use with stroke patients. *Age Ageing* 1983;**12**:166–70.
25. Duro D, Simões MR, Ponciano E, et al. Validation studies of the portuguese experimental version of the Montreal Cognitive Assessment (MoCA): confirmatory factor analysis. *J Neurol* 2010;**257**:728–34.
26. Nasreddine ZS, Phillips NA, Bédirian V, et al. The Montreal Cognitive Assessment, MoCA: a brief screening tool for mild cognitive impairment. *J Am Geriatr Soc* 2005;**53**:695–9.
27. Freitas S, Simões MR, Alves L, et al. Montreal Cognitive Assessment (MoCA): normative study for the portuguese population. *J Clin Exp Neuropsychol* 2011;**33**:989–96.
28. Ruano L, Sousa A, Severo M, et al. Development of a self-administered web-based test for longitudinal cognitive assessment. *Sci Rep* 2016;**6**:19114.
29. Schmand B, Jonker C, Hooijer C, et al. Subjective memory complaints may announce dementia. *Neurology* 1996;**46**:121–5.
30. Herdman M, Gudex C, Lloyd A, et al. Development and preliminary testing of the new five-level version of EQ-5D (EQ-5D-5L). *Qual Life Res* 2011;**20**:1727–36.
31. Wilson JTL, Hareendran A, Grant M, et al. Improving the assessment of outcomes in stroke: use of a structured interview to assign grades on the modified Rankin Scale. *Stroke* 2002;**33**:2243–6.
32. Broderick JP, Adeoye O, Elm J. Evolution of the Modified Rankin Scale and its use in future stroke trials. *Stroke* 2017;**48**:2007–12.
33. Kim D. The effects of hand strength on upper extremity function and activities of daily living in stroke patients, with a focus on right hemiplegia. *J Phys Ther Sci* 2016;**28**:2565–7.
34. Bohannon RW, Peolsson A, Massy-Westropp N, et al. Reference values for adult grip strength measured with a jamar dynamometer: a descriptive meta-analysis. *Physiotherapy* 2006;**92**:11–5.
35. Sousa-Santos AR, Amaral TF. Differences in handgrip strength protocols to identify sarcopenia and frailty - A systematic review. *BMC Geriatr* 2017;**17**:238.
36. Podsiadlo D, Richardson S. The Timed “Up & Go”: A test of basic functional mobility for frail elderly persons. *J Am Geriatr Soc* 1991;**39**:142–8.
37. Springer BA, Marin R, Cyhan T, et al. Normative values for the unipedal stance test with eyes open and closed. *J Geriatr Phys Ther* 2007;**30**:8–15.
38. Langhammer B, Stanghelle JK. Senior fitness test; a useful tool to measure physical fitness in persons with acquired brain injury. *Brain Inj* 2019;**33**:183–8.
39. Langhammer B, Stanghelle JK. The Senior Fitness Test. *J Physiother* 2015;**61**:163.
40. Guralnik JM, Simonsick EM, Ferrucci L, et al. A short physical performance battery assessing lower extremity function: association with self-reported disability and prediction of mortality and nursing home admission. *J Gerontol* 1994;**49**:M85–94.
41. Araújo F, Oliveira A, Pinto C, et al. [Validation of the Barthel Index in a sample of non-institutionalized elderly]. *Revista Portuguesa de Saúde Pública* 2007;**25**:59–66.
42. Teare MD, Dimairo M, Shephard N, et al. Sample size requirements to estimate key design parameters from external pilot randomised controlled trials: a simulation study. *Trials* 2014;**15**.
43. Kirk H, Kersten P, Crawford P, et al. The cardiac model of rehabilitation for reducing cardiovascular risk factors post transient ischaemic attack and stroke: a randomized controlled trial. *Clin Rehabil* 2014;**28**:339–49.

For peer review only - http://bmjopen.bmj.com/site/about/guidelines.xhtml

44. Ramos E, Lopes C, Barros H. Investigating the effect of nonparticipation using a population-based case-control study on myocardial infarction. *Ann Epidemiol* 2004;**14**:437–41.
45. World Medical Association. World Medical Association Declaration of Helsinki: ethical principles for medical research involving human subjects. *JAMA* 2013;**310**:2191–94.

For peer review only

For peer review only - http://bmjopen.bmj.com/site/about/guidelines.xhtml

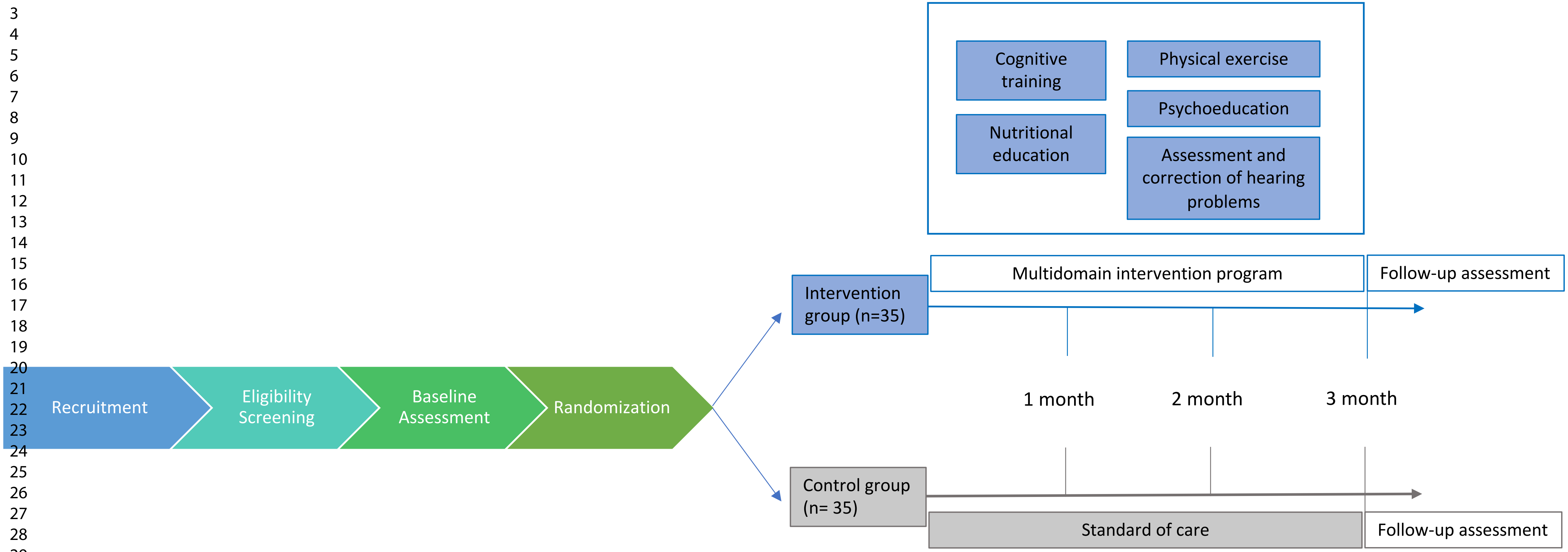
Cognitive training
Physical exercise
Psychoeducation
Nutritional education
Assessment and correction of hearing problems
Multidomain intervention program
Follow-up assessment
Intervention group (n=35)
Recruitment
Eligibility Screening
Baseline Assessment
Randomization
1 month
2 month
3 month
Control group (n= 35)
Standard of care
Follow-up assessment

SPIRIT
STANDARD PROTOCOL ITEMS: RECOMMENDATIONS FOR INTERVENTIONAL TRIALS

SPIRIT 2013 Checklist: Recommended items to address in a clinical trial protocol and related documents*

Section/item	Item No	Description	Addressed on page number
**Administrative information**			
Title	1	Descriptive title identifying the study design, population, interventions, and, if applicable, trial acronym	1
Trial registration	2a	Trial identifier and registry name. If not yet registered, name of intended registry	3
	2b	All items from the World Health Organization Trial Registration Data Set	Trial register NCT05369637
Protocol version	3	Date and version identifier	n/a
Funding	4	Sources and types of financial, material, and other support	22
Roles and responsibilities	5a	Names, affiliations, and roles of protocol contributors	1, 22
	5b	Name and contact information for the trial sponsor	22
	5c	Role of study sponsor and funders, if any, in study design; collection, management, analysis, and interpretation of data; writing of the report; and the decision to submit the report for publication, including whether they will have ultimate authority over any of these activities	22
	5d	Composition, roles, and responsibilities of the coordinating centre, steering committee, endpoint adjudication committee, data management team, and other individuals or groups overseeing the trial, if applicable (see Item 21a for data monitoring committee)	n/a

For peer review only

**Introduction**			
Background and rationale	6a	Description of research question and justification for undertaking the trial, including summary of relevant studies (published and unpublished) examining benefits and harms for each intervention	5, 6
	6b	Explanation for choice of comparators	9,10
Objectives	7	Specific objectives or hypotheses	6
Trial design	8	Description of trial design including type of trial (eg, parallel group, crossover, factorial, single group), allocation ratio, and framework (eg, superiority, equivalence, noninferiority, exploratory)	7
**Methods: Participants, interventions, and outcomes**			
Study setting	9	Description of study settings (eg, community clinic, academic hospital) and list of countries where data will be collected. Reference to where list of study sites can be obtained	7
Eligibility criteria	10	Inclusion and exclusion criteria for participants. If applicable, eligibility criteria for study centres and individuals who will perform the interventions (eg, surgeons, psychotherapists)	7, 8
Interventions	11a	Interventions for each group with sufficient detail to allow replication, including how and when they will be administered	9 to 12
	11b	Criteria for discontinuing or modifying allocated interventions for a given trial participant (eg, drug dose change in response to harms, participant request, or improving/worsening disease)	19
	11c	Strategies to improve adherence to intervention protocols, and any procedures for monitoring adherence (eg, drug tablet return, laboratory tests)	10
	11d	Relevant concomitant care and interventions that are permitted or prohibited during the trial	9,10
Outcomes	12	Primary, secondary, and other outcomes, including the specific measurement variable (eg, systolic blood pressure), analysis metric (eg, change from baseline, final value, time to event), method of aggregation (eg, median, proportion), and time point for each outcome. Explanation of the clinical relevance of chosen efficacy and harm outcomes is strongly recommended	12 to 16
Participant timeline	13	Time schedule of enrolment, interventions (including any run-ins and washouts), assessments, and visits for participants. A schematic diagram is highly recommended (see Figure)	9, 10

For peer review only - http://bmjopen.bmj.com/site/about/guidelines.xhtml

Sample size	14	Estimated number of participants needed to achieve study objectives and how it was determined, including clinical and statistical assumptions supporting any sample size calculations	17
Recruitment	15	Strategies for achieving adequate participant enrolment to reach target sample size	10
**Methods: Assignment of interventions (for controlled trials)**			
Allocation:			
Sequence generation	16a	Method of generating the allocation sequence (eg, computer-generated random numbers), and list of any factors for stratification. To reduce predictability of a random sequence, details of any planned restriction (eg, blocking) should be provided in a separate document that is unavailable to those who enrol participants or assign interventions	8
Allocation concealment mechanism	16b	Mechanism of implementing the allocation sequence (eg, central telephone; sequentially numbered, opaque, sealed envelopes), describing any steps to conceal the sequence until interventions are assigned	8
Implementation	16c	Who will generate the allocation sequence, who will enrol participants, and who will assign participants to interventions	8
Blinding (masking)	17a	Who will be blinded after assignment to interventions (eg, trial participants, care providers, outcome assessors, data analysts), and how	16
	17b	If blinded, circumstances under which unblinding is permissible, and procedure for revealing a participant's allocated intervention during the trial	16
**Methods: Data collection, management, and analysis**			
Data collection methods	18a	Plans for assessment and collection of outcome, baseline, and other trial data, including any related processes to promote data quality (eg, duplicate measurements, training of assessors) and a description of study instruments (eg, questionnaires, laboratory tests) along with their reliability and validity, if known. Reference to where data collection forms can be found, if not in the protocol	12 to 17
	18b	Plans to promote participant retention and complete follow-up, including list of any outcome data to be collected for participants who discontinue or deviate from intervention protocols	10,18

For peer review only - http://bmjopen.bmj.com/site/about/guidelines.xhtml

Data management	19	Plans for data entry, coding, security, and storage, including any related processes to promote data quality (eg, double data entry; range checks for data values). Reference to where details of data management procedures can be found, if not in the protocol	19, 20
Statistical methods	20a	Statistical methods for analysing primary and secondary outcomes. Reference to where other details of the statistical analysis plan can be found, if not in the protocol	17
	20b	Methods for any additional analyses (eg, subgroup and adjusted analyses)	n/a
	20c	Definition of analysis population relating to protocol non-adherence (eg, as randomised analysis), and any statistical methods to handle missing data (eg, multiple imputation)	17
**Methods: Monitoring**			
Data monitoring	21a	Composition of data monitoring committee (DMC); summary of its role and reporting structure; statement of whether it is independent from the sponsor and competing interests; and reference to where further details about its charter can be found, if not in the protocol. Alternatively, an explanation of why a DMC is not needed	19
	21b	Description of any interim analyses and stopping guidelines, including who will have access to these interim results and make the final decision to terminate the trial	n/a
Harms	22	Plans for collecting, assessing, reporting, and managing solicited and spontaneously reported adverse events and other unintended effects of trial interventions or trial conduct	19
Auditing	23	Frequency and procedures for auditing trial conduct, if any, and whether the process will be independent from investigators and the sponsor	n/a
**Ethics and dissemination**			
Research ethics approval	24	Plans for seeking research ethics committee/institutional review board (REC/IRB) approval	2
Protocol amendments	25	Plans for communicating important protocol modifications (eg, changes to eligibility criteria, outcomes, analyses) to relevant parties (eg, investigators, REC/IRBs, trial participants, trial registries, journals, regulators)	19

For peer review only - http://bmjopen.bmj.com/site/about/guidelines.xhtml

Consent or assent	26a	Who will obtain informed consent or assent from potential trial participants or authorised surrogates, and how (see Item 32)	8
	26b	Additional consent provisions for collection and use of participant data and biological specimens in ancillary studies, if applicable	n/a
Confidentiality	27	How personal information about potential and enrolled participants will be collected, shared, and maintained in order to protect confidentiality before, during, and after the trial	19, 20
Declaration of interests	28	Financial and other competing interests for principal investigators for the overall trial and each study site	22
Access to data	29	Statement of who will have access to the final trial dataset, and disclosure of contractual agreements that limit such access for investigators	19, 20
Ancillary and post-trial care	30	Provisions, if any, for ancillary and post-trial care, and for compensation to those who suffer harm from trial participation	n/a
Dissemination policy	31a	Plans for investigators and sponsor to communicate trial results to participants, healthcare professionals, the public, and other relevant groups (eg, via publication, reporting in results databases, or other data sharing arrangements), including any publication restrictions	20,21
	31b	Authorship eligibility guidelines and any intended use of professional writers	n/a
	31c	Plans, if any, for granting public access to the full protocol, participant-level dataset, and statistical code	n/a
**Appendices**			
Informed consent materials	32	Model consent form and other related documentation given to participants and authorised surrogates	Submitted in the revision
Biological specimens	33	Plans for collection, laboratory evaluation, and storage of biological specimens for genetic or molecular analysis in the current trial and for future use in ancillary studies, if applicable	n/a

*It is strongly recommended that this checklist be read in conjunction with the SPIRIT 2013 Explanation & Elaboration for important clarification on the items. Amendments to the protocol should be tracked and dated. The SPIRIT checklist is copyrighted by the SPIRIT Group under the Creative Commons "Attribution-NonCommercial-NoDerivs 3.0 Unported" license.

For peer review only - http://bmjopen.bmj.com/site/about/guidelines.xhtml
